# Estimating health-state utility values for family-caregivers of patients with Duchenne muscular dystrophy using time trade-off valuation

**DOI:** 10.1186/s41687-026-01055-8

**Published:** 2026-04-10

**Authors:** Oktawia Borecka, Samuel Llewellyn, Ione Woollacott, Lucy Richardson, Alasdair Fellows, Jack Lawrence, Catherine Bottomley, Alice M. Biggane

**Affiliations:** 1 Vitaccess, 2nd Floor Nucleus House, 2 Lower Mortlake Road, Richmond, London, TW9 2JA UK; 2 Pfizer Ltd., Tadworth, UK

## Abstract

**Objectives:**

Duchenne muscular dystrophy (DMD) is a rare, progressive neuromuscular disease. Long-term care is primarily provided by unpaid family-caregivers. This time trade-off (TTO) study aimed to generate family-caregiver utility values associated with different stages of DMD.

**Methods:**

Eight vignettes were developed to reflect the experience of caring for patients with DMD across health states (HS) defined in the Project HERCULES model. Vignettes were informed by literature and input from family-caregivers and healthcare professionals. TTO interviews were conducted online with members of the UK general public. Participants reviewed all vignettes and completed HS ranking, visual analog scale (VAS) ratings, and TTO valuation tasks.

**Results:**

200 participants (mean age 44.0 years, 51% female) completed interviews. Mean utility scores were highest for HS1 (early ambulatory: 0.717) and lowest for HS8 (no hand-to-mouth function, full-time ventilation: 0.477). VAS scores showed a similar pattern, with HS1 scoring 65.49 and HS8 scoring 31.665.

**Conclusions:**

This study highlights the increasing burden on family-caregivers as DMD progresses, with declining health-related quality of life across HS. The resulting utility values support inclusion of family-caregiver outcomes in future DMD cost-effectiveness models.

**Supplementary Information:**

The online version contains supplementary material available at 10.1186/s41687-026-01055-8.

## Introduction

Duchenne muscular dystrophy (DMD) is a rare, genetic, terminal neuromuscular disease, characterized by progressive muscular degeneration and severe multisystem complications [1, 2]. Estimates of DMD incidence range from 1 in 3,800 to 1 in 6,200 live births, almost exclusively affecting boys, with disruptions to daily life beginning as early as three years of age [2]. The condition results in a broad spectrum of physical and psycho-social consequences not only for the patient but also, for their caregiver(s) [3].

The long-term care of patients with DMD is predominantly provided at home by informal and unpaid family members [4], most often by their parents, who are known as “family-caregivers” [5]. As patients with DMD transition to a state of total dependency due to disease progression, the burden experienced by family-caregivers of patients with DMD can be extensive and debilitating [6]. Prior research suggests family-caregivers suffer in terms of their mental and physical health [7], due to the emotional and social support that they provide alongside their required assistance with basic activities of daily living. Caring for a patient with DMD can be financially burdensome, with notable proportions of family-caregivers leaving the workforce completely or reporting significant reductions in their work productivity due to their caring responsibilities [4].

There is a distinct lack of effective treatments for DMD. Current management of the disease consists of efforts to slow disease progression through the use of corticosteroids and a focus on improving health-related quality of life (HRQoL) via preservation of muscle strength and function to support mobility and delay complications [8]. Emerging technologies and interventions are focused on more effective steroid treatments and progressing genetic therapies [9–11]. However, before patients can access these emerging new treatments, they require clinical and economic evaluations, by a range of decision makers. There is a growing consensus that family-caregivers should be considered, alongside patients, in such evaluations, ensuring their direct health and social impacts and associated spillover effects [12–14] are also assessed.

Cost-effectiveness analyses (CEA) are widely used by many health technology assessment (HTA) agencies as a central tool for evaluating new treatments. These analyses typically account for the treatment’s impact on HRQoL using preference-based measures which allow for the calculation of utility values. Utility values reflect the strength of preference that individuals (or populations) assign to different health states (HS), anchored at 0 for being dead and 1 for full health. These values are used to calculate quality-adjusted life years (QALYs) Utility scores can also take negative values, representing HS considered worse than dead.

When caregiving imposes a substantial burden on families, utility values for family-caregivers may also be included, alongside those for patients, to capture the broader impact of disease and treatment [15, 16].

Utility values are often derived from generic preference-based measures intended for use across a wide range of health conditions [17]. However, these measures may not fully capture all relevant aspects of HRQoL, particularly for individuals affected by rare, progressive diseases [18], and their family-caregivers [19]. In the context of caregiving for DMD, such instruments may be limited, as they often overlook domains, such as disruption to work or education, and the broad emotional toll of providing long-term care for someone with a progressive, life-limiting condition [20, 21]. In these instances, one alternative recommended by the National Institute for Health and Care Excellence (NICE) – the national HTA body of England – is a vignette study [17]. Vignettes describe the impacts of a condition on HRQoL from the perspective of either the patients or the family-caregivers. Utility values are then generated by valuing these vignettes in techniques such as time trade off (TTO) methods [22].

While family-caregiver utilities for DMD have been reported in the literature [23, 24], these values pre-date the development of the recent HS model (Table 1) by Project HERCULES [25, 26], Resultingly, they lack sufficient granularity to differentiate fully between the eight defined HS from the model. Furthermore, the family-caregiver utilities reported by Landfeldt et al. [23] were derived from the EQ-5D, and given the generic nature of the instrument, these values may not capture the complexity and nuance of HRQoL impacts experienced by family-caregivers of patients with DMD [27].

**Table 1:** Summary of DMD HS names as defined by Project HERCULES [25, 26]

HS abbreviation	Full DMD HS name
HS1	Early ambulatory
HS2	Late ambulatory
HS3	Transfer stage
HS4	HTMF, no vent
HS5*	No HTMF, no vent
HS6*	HTMF, night vent
HS7	No HTMF, night vent
HS8	No HTMF, full vent

Abbreviations: HTMF = hand-to-mouth function, no vent = no ventilation required, night vent = nighttime ventilation required, full vent = full time ventilation required

*Patients with DMD can enter HS5 or HS6 from HS4 only and will then progress directly from either to HS7

Thus, this study aims to complement the existing evidence base by focusing on the detailed and condition-specific impacts of DMD on family-caregivers. To address this, we used a vignette-based TTO approach aligned with the Project HERCULES model to provide detailed, condition-specific insights for use in future health economic evaluations. This method allowed us to reflect nuanced aspects of the family-caregiver experience, such as emotional strain, educational disruption, and employment challenges [20, 22].

## Methods

### Study design

This was a non-interventional, cross-sectional, interview-based TTO vignette study. Adult members of the United Kingdom (UK) general population participated in structured interviews during September - December 2023 via one-on-one web conference, in which they were asked to rate a series of vignettes describing caring for a patient with DMD in each of the eight Project HERCULES model-defined HS, using the TTO valuation technique.

Early in the study design phase, Patient and Public Involvement (PPI) contributors were engaged to inform the overall study concept, vignette structure, and framing. The patient advocacy group (PAG) Duchenne UK (DUK) provided experiential input regarding relevant, terminology, and family-caregiver-centered framing. In parallel, a health economics key opinion leader (KOL) advised on methodological considerations for vignette design and the overall valuation approach. These activities constituted PPI and expert consultation, not data-generating research.

All individuals and organizations contributing at this stage were UK-based. PPI contributors and the KOL were compensated according to UK fair market value guidance.

### Vignette development

**Fig. 1:**
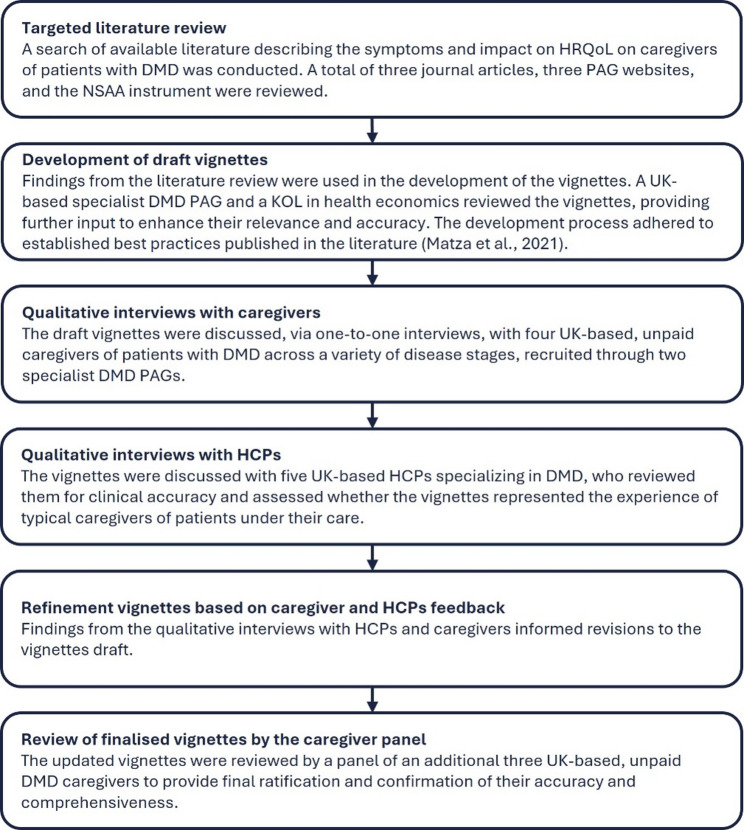
Flow diagram of methodological steps taken to develop the vignettes of family-caregivers of patients with DMD. Abbreviations: DMD: Duchenne muscular dystrophy, HCP: healthcare professional, HRQoL: health-related quality of life, KOL: key opinion leader, NSAA: North Star Ambulatory Assessment, PAG: patient advocacy group

Following an evidence-based approach, utilizing multiple high-quality data sources including relevant published literature and DMD PAG websites, eight vignettes (HS descriptions) were drafted, representing family-caregiver experiences across the eight HS of DMD defined in the Project HERCULES model [25, 26]. A schematic of the development process is shown in Fig. 1.

Vignette development involved qualitative research with research participants (distinct from PPI contributors). All participants provided informed consent.

All draft vignettes were first evaluated through one-to-one interviews with four unpaid family-caregivers of patients with DMD, representing a variety of disease stages, recruited through DUK and Pathfinders Neuromuscular Alliance (PNA). Their feedback focused on clarity, realism and completeness of the vignettes.

Five healthcare professionals (HCPs) specializing in DMD (three consultant pediatric neurologists and two neuromuscular care advisors) reviewed the vignettes for clinical accuracy and assessed whether they reflected the experience of typical family-caregivers of patients under their care.

Revisions to the vignettes were made following analysis of feedback from both family-caregiver and HCP participants. Where feedback diverged, these points were reviewed within the study team and, where appropriate, the vignettes were refined to incorporate both perspectives to ensure balanced representation. In instances of divergent feedback, caregiver perspectives guided the final wording to align with the study’s focus on caregiver utilities, with HCP input ensuring that descriptions remained clinically accurate.

The revised vignettes were subsequently reviewed in follow-up qualitative interviews with an additional three unpaid DMD family-caregivers recruited via PNA. Their input provided independent validation of the accuracy and comprehensiveness of the updated vignettes, informing final refinements.

At each stage, changes were incorporated in the vignettes based on the qualitative data collected. The study sponsor remained blinded to participant identities. To minimize potential bias, research participants were initially blinded to the identity of the study sponsor; this information was disclosed to them at the end of the interview. All research participants were compensated for their time in accordance with UK fair market value guidance. Participants were informed that they could stop their interviews at any time without consequence.

Alongside the eight DMD HS family-caregiver vignettes, two additional vignettes were developed: one representing a person in full health who had no DMD-related caregiving responsibilities, and another representing the state of being dead. A generic dead vignette was sourced from literature, while a full health vignette was developed by the study team based on their research. Our description of full health focused on the absence of family-caregiver-specific impacts and served as a standard anchor point within this methodological context. This aligns with accepted practice in condition-specific vignette valuation studies. As noted in Hall et al. [28] “A measure of health status (health utilities) are conventionally anchored at 0 (dead) and 1 (full health)”, and the TTO method is widely used within this framework. Similarly, Martin et al. [29] also employed an anchoring approach in their vignette valuation work on a rare disease. The ten vignettes are available in Supplementary Materials.

### Ethical approvals

Ethics approval for this study was granted by a central independent review board (Solutions IRB, Yarnell, AZ, US). Informed consent was obtained electronically from all study participants.

### Participants

The study enrolled adult members of the UK general population. Participants were recruited by a specialist recruitment agency (Global Perspectives, Reading, UK), via a combination of social media targeting, the agency’s existing research databases/panels, and networking/snowballing. Recruitment was monitored in an attempt to align the characteristics of the sample with UK population norms for age, sex, ethnicity, country of residence, employment status, education, and marital status [30–35].

A target sample size of 200 participants was set, which aligned with similar published TTO studies [28, 29]. No formal sample size calculations were performed as the study was not designed to test a specific hypothesis. Prior to participation, potential participants were provided with detailed study information via email and were asked to electronically sign an informed consent document. Participants received a £35 e-gift card for their time following completion of the interview. The study sponsor remained blinded to the identity of participants throughout the study. To minimize potential bias, research participants were initially blinded to the identity of the study sponsor; this information was disclosed to them on completion of their interviews.

To be included in the study, participants had to meet the following criteria: (1) 18 years or older, (2) resident in the UK (England, Scotland, Wales, or Northern Ireland), (3) able and willing to participate in an interview in English, and (4) able and willing to provide informed consent to participate. Individuals with acute illness or cognitive impairment deemed by the investigator to interfere with study requirements were excluded.

### Data collection

Study materials and conduct were informed by the team’s prior experience, the disease area, and learnings from other online TTO interviews [36]. The pilot with 20 UK general public participants assessed the interview design and materials. As no issues were identified, they were included in the main study sample.

All interviews were conducted online via Microsoft Teams by trained interviewers. Each interview lasted approximately 60 min, and was scheduled at a time convenient for participants. Participants were informed that they could stop the interview at any time without consequence. Interviews were not recorded; instead, interviewers entered results for each component (ranking, rating, TTO exercises) directly into a spreadsheet. In total, nine interviewers conducted the one-on-one interviews (range = 9—52 per interviewer, mean = 26.1).

### Interviews

Participants received the ten vignettes by email 24 h before the interview. To minimize bias, vignettes were presented in random order and labelled with symbols. For the visual analogue scale (VAS) task, participants rated HS in the order shown in their version (one of four variants). For the TTO task, interviewers randomized HS order during the interview to reduce potential order effects. Participants were encouraged, but not required, to read the vignettes beforehand.

Participants were informed that they would not be made aware of the name of the condition being investigated. The vignettes described the condition broadly to avoid bias [37] and included generic HRQoL domains for a full depiction of lived experience. During the interview (script available in Supplementary Materials), participants answered sociodemographic questions and completed three tasks: vignette ranking, rating, and TTO valuation. Participants ranked the ten vignettes from best to worst using an on-screen aid, with no ties allowed, to familiarize them with the HS and the valuation process. They then rated each vignette on a 100-point VAS (full health = 100). Interviews were terminated if participants repeatedly ranked late non-ambulatory HS above ambulatory HS, even after interviewers reiterated the task instructions.

**Fig. 2:**
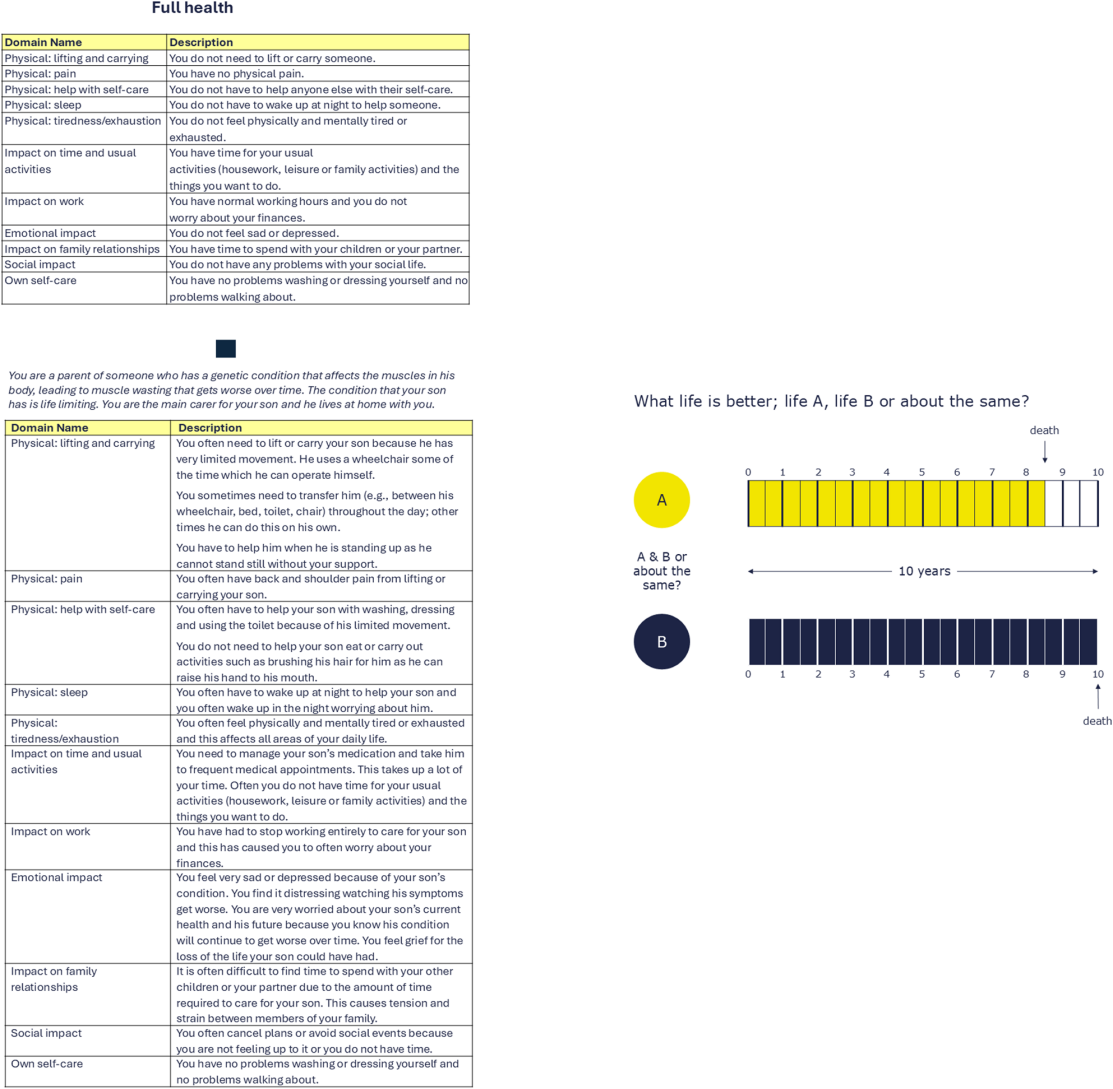
Example of the TTO exercise seen by participants

The TTO task used a composite approach via an on-screen TTO board to elicit utility values (Fig. 2). Using the ping-pong method, participants chose between living up to 10 years in full health or 10 years in a specified HS, with the full health time adjusted in 6-month increments until a point of indifference was reached. Utility values were calculated using the formula: utility = years in full health/years in HS [38]. For HS considered worse than dead, the lead-time TTO (LT-TTO) method was applied. In this task, participants chose between 10 years in full health followed by immediate death, or 10 years in full health followed by 10 years in the HS, then immediate death. Utility values for these states were calculated using the formula: utility = (years in full health – years of lead-time)/years in HS) [38]. Utilities were censored at -1 to limit the minimum value for states considered worse than dead. A 10-year time horizon was applied in both TTO and LT-TTO tasks, reflecting common practice in valuation studies for chronic conditions [39, 40]. During data collection, interviewers followed a structured protocol, and where participants provided repeated valuations without apparent differentiation, they were prompted to confirm their understanding of the task. The TTO methodology aligned with international and UK-specific HS valuation protocols [41, 42].

### Variables

Data that were collected during the interviews included participants’ socio-demographics (age, sex, country, ethnicity, employment status, education, marital status, total annual household income, caring or parenting responsibilities and number of children or dependents, if applicable) and vignette valuation data (ranking exercise for each of the 10 HS; VAS score (0-100) for each of the eight DMD family-caregiver HS and full health and dead HS; and TTO interview exercise score (1.00 to -1.00) for each of the eight DMD family-caregiver HS).

### Statistical analysis

Vignette valuation data were stored securely and analyzed using descriptive statistics. Continuous variables were summarized by number, mean, standard deviation (SD), median, interquartile range (IQR), minimum, and maximum; and categorical variables by number, frequency, and proportion.

In addition to the descriptive summaries, we conducted pairwise paired t-tests to compare mean VAS and utility scores between HS within participants. To control the family-wise error rate across multiple comparisons, p-values were adjusted using the Holm correction. Accordingly, any adjusted p-value less than 0.05 was deemed statistically significant. These tests provided complimentary, within-respondent contrasts of HS alongside the regression analyses.

A mixed model of utility scores for the eight DMD family-caregiver HS (with ‘perfect health’ as the reference category) was fitted as a repeated-measures model. Sociodemographic variables were included as fixed effects, and the model was adjusted for age, sex, ethnicity, employment, income, country, marital status, education, and family-caregiver status. A unique participant identifier was included as a random intercept to account for within-participant correlation arising from repeated valuations. Some variables with sparse categories were collapsed to ensure sufficient cell sizes (e.g., ethnicity: White vs. non‑White; region: England vs. outside England). Other variables were retained in their original categorical forms to preserve conceptually meaningful distinctions that would be lost through further collapsing. As these covariates served as contextual variables rather than primary predictors of utility, this approach balanced parsimony with interpretability. Sensitivity checks using more simplified versions of these variables produced comparable results, supporting the robustness of the modelling strategy. A further sensitivity analysis was conducted excluding participants who provided identical utility scores across all HS levels (“non-traders”) to assess the robustness of the primary results.

All analyses were conducted in R version 4.3.2, using the lmer function from the lme4 package.

## Results

### Participants

This study enrolled 200 UK general public members, aiming to match UK demographics as closely as feasible (Table 2). The gender split was 51% female, 49% male, with an age profile broadly reflecting the UK. 98% of participants resided in England. 71% reported being parents or family-caregivers, most commonly caring for two children/dependents (29%), then one (22%). Only 9% cared for three or more, none more than four. The most common household income was £75,000+ (19%), followed by £25,000–£34,999 (16%).

Ten interviews were terminated by interviewers due to participants’ lack of understanding, and two were ended at participants’ request due to uncertainty about completing the tasks. These 12 were excluded and replaced to reach the 200-participant target.

**Table 2:** Demographic data of the study sample vs. the UK population

Demographic	Study sample (*N* = 200)	UK population
**Sex (%)**		
Female	102 (51.0%)	50.6%
Male	98 (49.0%)	49.4%
**Age (years)**		
Mean (SD)	44.0 (14.3)	—
Median (IQR)	42.0 (33.0–55.0)	40.7
Range	19–75	—
**Ethnicity**^**#**^ **(%)**		
White	156 (78.0%)	81.7%
Asian or Asian British	23 (11.5%)	9.3%
Black, Black British, Caribbean or African	12 (6.0%)	4.0%
Mixed or multiple ethnic groups	8 (4.0%)	2.9%
Other ethnic group	1 (0.5%)	2.1%
**Country of residence (%)**		
England	196 (98.0%)	84.3%
Wales	3 (1.5%)	4.6%
Scotland	1 (0.5%)	8.2%
Northern Ireland	0 (0.0%)	2.9%
**Employment status (%)**		
Full-time	80 (40.0%)	40.2%
Part-time	39 (19.5%)	17.0%
Retired	30 (15.0%)	21.6%
Unemployed	29 (14.5%)	3.4%
Student	12 (6.0%)	5.6%
Self-employed	9 (4.5%)	—
Other*	1 (0.5%)	12.1%
**Qualifications (%)** ^**†**^		
Qualifications (non-university)	115 (57.5%)	48.0%
Qualifications (university)	82 (41.0%)	33.8%
No formal qualifications	3 (1.5%)	18.2%
**Marital status (%)**		
Partner/married	108 (54.0%)	46.9%
Single	69 (34.5%)	37.9%
Divorced/separated/widowed	23 (11.5%)	15.2%
**Parent or family-caregiver status (%)**		
Yes	142 (71.0%)	—
No	58 (29.0%)	—
**Number of children or dependents under care (%)**		
1 child	44 (22.0%)	—
2 children	58 (29.0%)	—
3 children	12 (6.0%)	—
4 children	6 (3.0%)	—
5 or more children	0 (0.0%)	—
N/A^§^	80 (40.0%)	—
**Annual household income before tax (including any benefits)**		
Up to £4,999	6 (3.0%)	—
£5,000 to £14,999	16 (8.0%)	—
£15,000 to £24,999	22 (11.0%)	—
£25,000 to £34,999	31 (15.5%)	—
£35,000 to £44,999	24 (12.0%)	—
£45,000 to £54,999	19 (9.5%)	—
£55,000 to £64,999	16 (8.0%)	—
£65,000 to £74,999	18 (9.0%)	—
£75,000 and above	37 (18.5%)	—
Prefer not to answer	11 (5.5%)	—

Notes: Age and population data taken from Population Estimates for the UK, England, Wales, Scotland and Northern Ireland: mid-2021 [33]. Sex data taken from Overview of the UK population: January 2021 [35]. Ethnicity, employment status, and education level data taken from Ethnic Group, England and Wales: Census 2021 [34], Economic Activity Status, England and Wales: Census 2021 [32], and Education, England and Wales: Census 2021 [31], respectively. Marital status data obtained from Marriage and civil partnership status in England and Wales: Census 2021 [30]

^#^White ethnicity group included participants who defined themselves to be English/Welsh/Scottish/Northern Irish/British, Irish, Gypsy or Irish Traveller, any other White background; Asian: Indian, Pakistani, Bangladeshi, Chinese, any other Asian/Asian British background; Black, Black British, Caribbean or African: African, Caribbean, any other Black/Black British/Caribbean background; Mixed or multiple ethnic groups: White and Black Caribbean, White and Black African, White and Asian, any other Mixed or multiple ethnic groups background; Other ethnic group: Arab, any other ethnic group background

*The person was both retired and working part-time

^†^Qualifications (university) group included participants who completed level 4 qualifications or above; Qualifications (non-university) group included participants who completed level 1, 2, 3 or apprenticeship qualifications

^§^N/A refers to non-parents/family-caregivers and parents who no longer have dependents under care

### VAS scores

The mean VAS scores for each HS are presented in Table 3. Among the DMD family-caregiver vignettes, HS1 had the highest mean score (65.49, SD = 20.843), while HS8 had the lowest (31.665, SD = 17.784). Scores for HS3 (42.325, SD = 19.266) and HS4, (42.435, SD = 18.412) were very similar. Relative to HS4, subsequent states generally had lower mean scores: HS5 (38.13, SD = 20.761), HS6 (37.785, SD = 16.831) and HS7 (33.355, SD = 20.536). Mean (SD) VAS scores for the anchor states were 99.75 (3.535) for full health and 2.11 (9.111) for dead. Pairwise paired t-tests indicated that the following differences were statistically significant: HS1 vs. HS2-HS8; HS2 vs. HS3-HS8; HS3 vs. HS5-HS8; HS4 vs. HS5-HS8; HS5 vs. HS7-HS8; and HS6 vs. HS7-HS8 (all *p* < 0.05; see Supplementary Materials).

**Table 3:** Mean (SD) and Median (IQR) VAS scores as obtained from rating task evaluating vignettes detailing the progression of DMD across multiple domains, through eight DMD HS, carried out with members of the general public

Health state	Mean (SD)	Median (IQR)
Early ambulatory (HS1)	65.49 (20.843)	70 (53.75, 80)
Late ambulatory (HS2)	57.005 (18.518)	60 (45, 70)
Transfer stage (HS3)	42.325 (19.266)	40 (30, 50)
HTMF, no vent (HS4)	42.435 (18.412)	40 (30, 55)
No HTMF, no vent (HS5)	38.13 (20.761)	35 (20, 50)
HTMF, night vent (HS6)	37.785 (16.831)	38.5 (28.75, 50)
No HTMF, night vent (HS7)	33.355 (20.535)	30 (20, 45)
No HTMF, full vent (HS8)	31.665 (17.784)	30 (20, 40)

Note: Patients with DMD can enter HS5 or HS6 only and will then progress directly from either to HS7

### Utility scores

The mean utility scores (obtained from the TTO task) for each HS are presented in Table 4. Among the DMD family-caregiver vignettes, HS1 had the highest mean utility score (0.717, SD = 0.348) and HS8 had the lowest (0.477, SD = 0.476). The mean scores for HS5 (0.503, SD = 0.466) and HS6 (0.544, SD = 0.451) were similar, with HS6 slightly higher by 0.04. Because the clinical pathway allows transitions to either HS5 or HS6, this small difference is consistent with expectations and does not indicate a temporal pattern. The largest difference between adjacent states was HS2 vs. HS3 (0.097; 0.674, SD = 0.384 vs. 0.577, SD = 0.428), and this difference was statistically significant (*p* < 0.01). Pairwise paired t-tests also indicated statistically significant differences for: HS1 vs. HS3-HS8; HS2 vs. HS4-HS8; HS3 vs. HS6-HS8; HS4 vs. HS6-HS8; and HS5 vs. HS8 (all *p* < 0.05; see Supplementary Materials).

A total of 22 participants moved from the standard TTO to the LT-TTO approach for valuing at least one HS. Transitions to LT-TTO occurred most frequently for the more severe HS, including HS8 (*n* = 17), HS6 (*n* = 14), and HS5 (*n* = 13).

**Table 4:** Mean (SD) and Median (IQR) utility scores as obtained from TTO task evaluating vignettes detailing the progression of DMD across multiple domains, through eight DMD HS, carried out with members of the general public

Health state	Mean (SD)	Median (IQR)
Early ambulatory (HS1)	0.717 (0.348)	0.837 (0.625, 0.95)
Late ambulatory (HS2)	0.674 (0.384)	0.8 (0.525, 0.925)
Transfer stage (HS3)	0.577 (0.428)	0.725 (0.4, 0.85)
HTMF, no vent (HS4)	0.561 (0.417)	0.675 (0.375, 0.825)
No HTMF, no vent (HS5)	0.503 (0.466)	0.625 (0.3, 0.8)
HTMF, night vent (HS6)	0.544 (0.451)	0.675 (0.362, 0.85)
No HTMF, night vent (HS7)	0.506 (0.446)	0.613 (0.275, 0.85)
No HTMF, full vent (HS8)	0.477 (0.476)	0.613 (0.275, 0.8)

Note: HS5 or HS6 are mutually exclusive. Patients will then progress directly from either to HS7

### Regression-based analyses

The results of the regression-based analyses are presented in Table 5.

The results show that HS2 through HS8 each had a significant negative parameter estimate, indicating that participants reported significantly (p-value < 0.05) lower utility scores for all HS compared with HS1. The only other significant variable was being a family-caregiver with 3 or more dependents, which was associated with a higher reported utility score compared with non-family-caregivers.

Model assumptions were checked and indicated some deviation away from normality in the residuals, likely due to clustering of responses at the upper and lower ends of the TTO scale. However, this level of deviation was considered acceptable, and the model remains robust. The model fit was summarized using a marginal r-squared value of 12% and a conditional r-squared value of 78%.

Nineteen participants reported the same utility score across all HS (non-traders; median flatline value = 0.675). A sensitivity analysis excluding these 19 participants yielded results comparable to the main model: HS2-HS8 remained significantly lower than HS1, and the association between *≥* 3 dependents and higher utility (vs. non-parent/family-caregiver) was unchanged in direction and significance. Absolute changes in state-level means ranged from (0.003–0.051) across all states.

**Table 5:** Relationship between sociodemographic variables and utility scores

TTO score	Parameter estimate	Standard error	95% confidence interval	*p*-value
Intercept	0.71	0.176	(0.362,1.053)	< 0.001
**Health state**				
HS2 (Late ambulatory)	-0.04	0.021	(-0.084,-0.002)	0.042*
HS3 (Transfer stage)	-0.14	0.021	(-0.181,-0.099)	< 0.001*
HS4 (HTMF, no vent)	-0.16	0.021	(-0.197,-0.114)	< 0.001*
HS5 (No HTMF, no vent)	-0.17	0.021	(-0.214,-0.131)	< 0.001*
HS6 (HTMF, night vent)	-0.21	0.021	(-0.255,-0.172)	< 0.001*
HS7 (No HTMF, night vent)	-0.21	0.021	(-0.252,-0.169)	< 0.001*
HS8 (No HTMF, full vent)	-0.24	0.021	(-0.281,-0.199)	< 0.001*
**Age**				
Years	-0.01	0.003	(-0.013,0)	0.066
**Sex**				
Male	0.07	0.059	(-0.041,0.19)	0.209
**Country**				
Not England	-0.07	0.198	(-0.459,0.319)	0.723
**Ethnicity**				
Non-white	-0.02	0.071	(-0.159,0.119)	0.779
**Employment status**				
Part-time	0.03	0.079	(-0.123,0.188)	0.685
Self-employed	-0.04	0.139	(-0.316,0.228)	0.751
Student	-0.14	0.143	(-0.416,0.144)	0.343
Retired	0.03	0.121	(-0.204,0.271)	0.782
Other	0.01	0.412	(-0.797,0.819)	0.979
Unemployed	0.07	0.099	(-0.127,0.259)	0.505
**Household income (including any benefits)**				
£25,000 to £54,999	0.08	0.082	(-0.085,0.236)	0.359
£55,000 and above	0.10	0.093	(-0.078,0.287)	0.262
Prefer not to answer	0.19	0.136	(-0.074,0.46)	0.159
**Education**				
Non-university	0.10	0.068	(-0.038,0.229)	0.162
No formal qualifications	0.04	0.247	(-0.447,0.522)	0.879
**Marital status**				
Divorced/separated/widowed	-0.10	0.095	(-0.282,0.092)	0.32
Single	-0.01	0.083	(-0.17,0.154)	0.921
**Parent or family-caregiver status**				
Parent/family-caregiver, but no dependents	0.12	0.125	(-0.126,0.366)	0.341
Parent/family-caregiver with 1 dependent	0.12	0.085	(-0.042,0.29)	0.144
Parent/family-caregiver with 2 dependents	0.16	0.087	(-0.014,0.326)	0.073
Parent/family-caregiver with 3 + dependents	0.37	0.123	(0.128,0.611)	0.003*

Note: The following variables were used as reference levels: health state = HS1 (Early ambulatory), country=England, sex=female, ethnicity=white, employment=full-time, income level=<£25,000, education/qualification=university, marital status=partner/married, parent/family-caregiver status = non-parent/family-caregiver

*indicates significance of p-value < 0.05

## Discussion

This study aimed to generate UK-specific utility values for family-caregivers of patients with DMD in each of the eight DMD HS as defined by Project HERCULES [26]. In an approach consistent with best practice, vignettes were developed using multiple data sources, including desktop research, engagement with UK DMD PAGs, interviews with family-caregivers of patients with DMD, and HCPs specializing in DMD [17].

This study provides a unique and robust set of family-caregiver utility values demonstrating the HRQoL impact experienced by family-caregivers of patients with DMD across a variety of disease stages. The increasing impact on family-caregivers during the disease progression of DMD is reflected with higher mean utility values in ambulatory stages (HS1 = 0.717; HS2 = 0.674) compared with later, transfer (HS3 = 0.577) and non-ambulatory stages of the disease (HS4 = 0.561; HS5 = 0.503; HS6 = 0.544; HS7 = 0.506; HS8 = 0.477). VAS scores followed a similar pattern. These values reflect a downward trajectory in family-caregiver HRQoL, likely attributed to escalating caregiving demands as DMD progresses. Increasing complexity – including loss of ability to perform daily activities, mobility support, and ventilation needs – places growing strain on family-caregivers. These findings highlight the potential advantage of DMD therapies capable of postponing the transition to more advanced stages of the disease, thereby preserving a higher HRQoL for family-caregivers (and patients) for longer.

Comparison with UK general population norms provides important context for interpreting the utilities derived in this study. Mean utility values reported in the literature for individuals completing the EQ-5D in the general population are 0.867 (SD 0.194) for the EQ-5D-3 L [43], 0.856 (SD 0.173) for the EQ-5D-5 L using the van Hout et al. crosswalk [44], and 0.905 (SD 0.142) for the EQ-5D-5 L using the Devlin et al. valuation set [42]. In contrast, the family-caregiver-derived utility values in our study are substantially lower across all DMD HS. Even at the earliest, ambulatory stages (HS1: 0.717; HS2: 0.674), values fall below general population norms, with a marked decline seen in transfer and non-ambulatory stages (HS3 to HS8 ranging from 0.577 to 0.477). This gap between the values for DMD family-caregivers and general population highlights the significant impact of caregiving in DMD and supports the importance of condition-specific valuation methods in capturing this burden.

We are not the first to use the TTO method to generate utility values for DMD family-caregivers. Gallop et al. [24] have implemented a similar methodology, but based on the available literature, we conclude that their focus was on two non-ambulatory HS only. Hence, the layers of granularity and nuance associated with DMD disease progression over time (highlighted as important via the Project HERCULES model) have not previously been fully explored. Nevertheless, the utility values they reported for the two HS examined were broadly comparable with ours: Gallop et al. reported mean utilities of 0.639 and 0.393 for HS most similar to our HS4 and HS8, respectively, while the current study elicited 0.561 and 0.477 for these corresponding states.

While capturing and understanding family and family-caregiver health spillover effects are important components of understanding the burden of a disease or the potential impact of new interventions, these spillovers are frequently overlooked in healthcare evaluations [45], as highlighted by the SHEER task force [12], a global panel of 17 multi-disciplinary area experts. When family spillovers are included, SHEER recommends the use of adequate time horizons, primary data collection and ensuring transparency. Bodies such as NICE, who endorse the inclusion of family-caregiver data in HTA, where relevant, use utility values generated via generic preference-based measures such as the EQ-5D. This helps provide the aforementioned level of transparency with regard to HRQoL evidence [17]. In a healthcare system, where resources are finite, this type of standardization and transparency, which is transferable across a range of disease areas, is often viewed favorably [46].

However, questions exist over the suitability of the EQ-5D to meaningfully capture the full impact of DMD for patients and their family-caregivers, a sentiment echoed in other progressive disease areas [47, 48]. The values generated in our current study might indirectly provide further evidence of the EQ-5D lacking sufficient sensitivity [21, 22] for this population. In a previous study by Landfeldt et al. [23], the EQ-5D was used to assess DMD family-caregiver HRQoL across four HS, resulting in values ranging from 0.85 (early ambulatory) to 0.79 (late non-ambulatory). These differences were modest compared with those observed in our study. It is important to note, however, that the two approaches measure different constructs: Landfeldt et al. derived indirect HRQoL values using a generic measure focused solely on the caregiver’s own impaired health status, whereas our study used a direct vignette-based TTO approach that produces a composite valuation reflecting both impaired HRQoL and the presence or absence of caregiving responsibilities. Accordingly, differences between studies may reflect both methodological differences and the broader scope of the vignette descriptions. Furthermore, the EQ-5D may overlook or undervalue domains particularly salient for DMD family-caregivers – such as emotional strain (stress, anxiety, depression) [5, 49], physical demands (mobility assistance, personal care) [18], and financial and social pressures [50–52]. Finally, in a recent evidence appraisal of ataluren for the treatment of DMD [53], NICE noted methodological concerns with using absolute caregiver EQ-5D values from Landfeldt et al., expressing a preference for a disutility-based approach and consequently assessing family-caregiver burden qualitatively. This illustrates the importance of recognizing how methodological choices – whether direct or indirect, and how family-caregiver effects are anchored – shape what is being measured and therefore how family-caregiver utility data are interpreted and used in HTA.

Beyond methodological considerations, utility values are crucial for evaluating healthcare interventions, but they are unlikely to capture the full impact of disease or interventions on the HRQoL of the patients and family-caregivers who experience them. Through their qualitative interview and survey study, Rosero et al. [54] emphasized the value of including family-caregiver perspectives, particularly in pediatric conditions like DMD. Their study captured the physical, emotional, and logistical impacts on family-caregivers, revealing significant impacts in these areas. Integrating such qualitative insights together with utility values would provide a more comprehensive understanding of the real-life impact on family-caregivers. Additionally, including broader insights into rare diseases, beyond family-caregiver perspectives, can better assess the overall societal impact. A recent conference presentation [51] highlighted the potential family-caregiver benefits of a gene therapy for DMD, *delandistrogene moxeparvovec*, suggesting that it could reduce caregiving hours and income loss compared with standard care. This further emphasizes the potential economic benefits of advanced therapies for rare diseases, not necessarily captured by utility values alone.

The family-caregiver utility values derived in this study present several strengths. The utilization of a diverse range of stakeholders in the development of the DMD family-caregiver vignettes, including DMD PAG representatives, individuals with lived experience as family-caregivers of patients with DMD, and specialist DMD HCPs, was crucial. A rigorous and iterative approach was adopted by collaborating closely with these stakeholders to ensure the accuracy of the vignettes in depicting the family-caregiver experience across multiple domains. This process, aligned with industry guidance [55, 56], aimed to ensure that the results would be both representative and reliable by incorporating different perspectives. Furthermore, as discussed previously, a large sample (*n* = 200) of the general population was recruited, aligning with other published TTO studies [28, 29]. Research further supports the equivalence of online video interviews (used in this study) and in-person interviews in generating TTO values, with both methods demonstrating feasibility, acceptability, and high-quality data [57, 58].

A potential limitation of the study, and other vignette-based studies, is the challenge of capturing the full complexity of the family-caregiver experience within vignettes. The multidimensional nature of DMD caregiving makes it challenging to convey within brief public-facing descriptions. To ensure accuracy, DMD family-caregivers and specialist HCPs were involved throughout vignette development, highlighting the value of diverse stakeholder input in designing tools for reliable utility values.

Another limitation is that the vignettes depict male children with DMD, as female cases are rare but do occur. This focus also strengthens relevance and validity by reflecting the predominant clinical presentation. Future studies could explore potential differences in HRQoL and lived experience between males and females with DMD.

Efforts were made to ensure the sample was representative of the UK general public; however, a limitation of the study is that not all variables could be perfectly matched. This was partly due to demographic reporting limitations, such as widowed, divorced, or separated individuals potentially self-reporting as single. Compared with the UK general public, the sample had higher unemployment rates, fewer participants in the ‘other’ employment category, and underrepresentation of individuals without formal qualifications. Annual household income was not included in the analysis, as suitable national data for direct comparison were unavailable. Additionally, the sample showed limited geographical representativeness. Relatedly, some sociodemographic subgroups (e.g., certain employment or qualification categories) had relatively small sample sizes. Although sensitivity checks using more collapsed versions of these variables produced results consistent with the main model, future research with larger and more diverse caregiver samples would enable more precise estimation and a more granular examination of potential subgroup differences.

The family-caregiver vignettes for each DMD HS were anchored to a full health vignette that made no explicit reference to caregiving responsibilities (see Supplementary Materials). While it is not possible to fully eliminate personal interpretation during valuation, interviewers reminded participants to focus on the quality of life as described in the vignettes. Interviews that showed evidence of misunderstanding this instruction or other elements of interview tasks were terminated. As such, we have no reason to believe this issue substantially affected the data, and the large sample size further mitigates potential individual-level bias. Moreover, the consistency of model outcomes after excluding participants with flatlined responses further supports the robustness and reliability of the data produced.

The TTO tasks used the conventional framing of 10 years in the described state followed by immediate death. For caregiver vignettes, this yields a composite valuation of living as described – capturing both the respondent’s own HRQoL and the presence or absence of caregiving responsibilities – rather than the isolated effect of caregiving alone. We acknowledge that this constant 10‑year framing is a simplification for a progressive condition such as DMD and may not fully reflect evolving experiences (e.g., uncertainty or anticipatory grief), despite efforts to incorporate such elements within the vignette descriptions. DMD is a real, lived condition; the valuation exercise is intended not to reduce these experiences to numbers, but to provide a structured, transparent approach consistent with current methods. We explicitly acknowledge these constraints as study limitations, and future research could consider designs that capture progression or disentangle caregiving from personal HRQoL (e.g., staged vignettes or duration‑based preference elicitation).

The discussions with family-caregivers and HCPs during the development of the vignettes used in this study highlighted the need for further research exploring the impacts of DMD, on not only primary family-caregivers, but also on siblings, extended family members, friends, colleagues and the wider community. Understanding the full spectrum of the impact of DMD and its spillover effects can provide valuable insights into the necessity and potential value of novel DMD treatments.

## Conclusions

To our knowledge, this is the first study which has elicited utility values for family-caregivers of patients with DMD across all DMD HS as defined by Project HERCULES [26] using a TTO vignette valuation exercise with members of the UK general public. The results of this study could be used to support future HTA submissions of novel DMD treatments.

Our findings illustrate that the responsibility of caring for an individual with DMD can result in a significant burden and diminished HRQoL, with this impact having been valued as becoming more pronounced as the disease progresses. This highlights the potential benefit of DMD therapies delaying disease progression, ultimately extending higher HRQoL for both family-caregivers and patients.

## Supplementary Information

Below is the link to the electronic supplementary material.

Supplementary Material 1

Supplementary Material 2

Supplementary Material 3

Supplementary Material 4

Supplementary Material 5

## Data Availability

Aggregated study data are available on request by contacting Vitaccess.
