# Supplementary material for: Estimating health-state utility values for family-caregivers of patients with Duchenne muscular dystrophy using time trade-off valuation
Source: J Patient Rep Outcomes. 2026 Apr 10;10:58. doi: 10.1186/s41687-026-01055-8 (PMC13076743; doi:10.1186/s41687-026-01055-8)
Supplement: Supplementary file 1 — Supplementary Material 1 [file 41687_2026_1055_MOESM1_ESM.docx]

**Full health**

| Domain Name | Description |
| --- | --- |
| Physical: lifting and carrying | You do not need to lift or carry someone. |
| Physical: pain | You have no physical pain. |
| Physical: help with self-care | You do not have to help anyone else with their self-care. |
| Physical: sleep | You do not have to wake up at night to help someone. |
| Physical: tiredness/exhaustion | You do not feel physically and mentally tired or exhausted. |
| Impact on time and usual activities | You have time for your usual activities (housework, leisure or family activities) and the things you want to do. |
| Impact on work | You have normal working hours and you do not worry about your finances. |
| Emotional impact | You do not feel sad or depressed. |
| Impact on family relationships | You have time to spend with your children or your partner. |
| Social impact | You do not have any problems with your social life. |
| Own self-care | You have no problems washing or dressing yourself and no problems walking about. |

**Dead**

You are dead.

**Early ambulatory**

*You are a parent of someone who has a genetic condition that affects the muscles in his body, leading to muscle wasting that gets worse over time. The condition that your son has is life limiting. You are the main carer for your son and he lives at home with you.*

| Domain Name | Description |
| --- | --- |
| Physical: lifting and carrying | You **rarely** need to lift or carry your son because he can move about independently. |
| Physical: pain | You **rarely** have back or shoulder pain from lifting or carrying your son. |
| Physical: help with self-care | You **sometimes** have to help your son with washing, dressing and using the toilet because of his age.  You **do not need to help** your son eat or carry out activities such as brushing his hair for him as he can raise his hand to his mouth. |
| Physical: sleep | You **do not usually** **have to** wake up at night to help your son but you **often** wake up in the night worrying about him. |
| Physical: tiredness/exhaustion | You **sometimes** feel physically and mentally tired or exhausted and this affects all areas of your daily life. |
| Impact on time and usual activities | You need to manage your son’s medication and take him to frequent medical appointments. **This takes up a lot of your time.** **Sometimes** you do not have time for your usual activities (housework, leisure or family activities) and the things you want to do. |
| Impact on work | Depending on the support available to you, you might have **reduced your working hours** to care for your son and this has caused you to **sometimes** worry about your finances. |
| Emotional impact | You feel **very** sad or depressed because of your son’s condition. You feel shock and grief because you have been told that his condition is serious and life-limiting. You are **very** worried about your son’s current health and his future because you know his condition will get worse over time. |
| Impact on family relationships | It is **sometimes** difficult to find time to spend with your other children or your partner due to the amount of time required to care for your son. This causes tension and strain between members of your family. |
| Social impact | You **sometimes** cancel plans or avoid social events because you are not feeling up to it or you do not have time. |
| Own self-care | You have no problems washing or dressing yourself and no problems walking about. |

**Late ambulatory**

*You are a parent of someone who has a genetic condition that affects the muscles in his body, leading to muscle wasting that gets worse over time. The condition that your son has is life limiting. You are the main carer for your son and he lives at home with you.*

| **Domain Name** | **Description** |
| --- | --- |
| Physical: lifting and carrying | You **often** need to lift or carry your son because he has some limited movement. |
| Physical: pain | You **sometimes** have back and shoulder pain from lifting or carrying your son. |
| Physical: help with self-care | You **sometimes** have to help your son with washing, dressing and using the toilet because of his limited movement.  You **do not need to help** your son eat or carry out activities such as brushing his hair for him as he can raise his hand to his mouth. |
| Physical: sleep | You **sometimes** have to wake up at night to help your son and you **often** wake up in the night worrying about him. |
| Physical: tiredness/exhaustion | You **sometimes** feel physically and mentally tired or exhausted and this affects all areas of your daily life. |
| Impact on time and usual activities | You need to manage your son’s medication and take him to frequent medical appointments. **This takes up a lot of your time**. **Sometimes** you do not have time for your usual activities (housework, leisure or family activities) and the things you want to do. |
| Impact on work | Depending on the support available to you, you might have **reduced your working hours** to care for your son and this has caused you to **sometimes** worry about your finances. |
| Emotional impact | You feel **very** sad or depressed because of your son’s condition. You feel shock and grief because you have been told that his condition is serious and life-limiting. You are **very** worried about your son’s current health and his future because you know his condition will get worse over time. |
| Impact on family relationships | It is **sometimes** difficult to find time to spend with your other children or your partner due to the amount of time required to care for your son. This causes tension and strain between members of your family. |
| Social impact | You **often** cancel plans with your friends or avoid social events because you are not feeling up to it or you do not have time. |
| Own self-care | You have no problems washing or dressing yourself and no problems walking about. |

**Transfer stage**

*You are a parent of someone who has a genetic condition that affects the muscles in his body, leading to muscle wasting that gets worse over time. The condition that your son has is life limiting. You are the main carer for your son and he lives at home with you.*

| Domain Name | Description |
| --- | --- |
| Physical: lifting and carrying | You **often** need to lift or carry your son because he has very limited movement. He uses a wheelchair some of the time which he can operate himself.  You **sometimes** need to transfer him (e.g., between his wheelchair, bed, toilet, chair) throughout the day; other times he can do this on his own.  You have to help him when he is standing up as he cannot stand still without your support. |
| Physical: pain | You **often** have back and shoulder pain from lifting or carrying your son. |
| Physical: help with self-care | You **often** have to help your son with washing, dressing and using the toilet because of his limited movement.  You **do not need to help** your son eat or carry out activities such as brushing his hair for him as he can raise his hand to his mouth. |
| Physical: sleep | You **often** have to wake up at night to help your son and you **often** wake up in the night worrying about him. |
| Physical: tiredness/exhaustion | You **often** feel physically and mentally tired or exhausted and this affects all areas of your daily life. |
| Impact on time and usual activities | You need to manage your son’s medication and take him to frequent medical appointments. **This takes up a lot of your time.** **Often** you do not have time for your usual activities (housework, leisure or family activities) and the things you want to do. |
| Impact on work | You have had to **stop working** entirely to care for your son and this has caused you to **often** worry about your finances. |
| Emotional impact | You feel **very** sad or depressed because of your son’s condition. You find it distressing watching his symptoms get worse. You are **very** worried about your son’s current health and his future because you know his condition will continue to get worse over time. You feel grief for the loss of the life your son could have had. |
| Impact on family relationships | It is **often** difficult to find time to spend with your other children or your partner due to the amount of time required to care for your son. This causes tension and strain between members of your family. |
| Social impact | You **often** cancel plans or avoid social events because you are not feeling up to it or you do not have time. |
| Own self-care | You have no problems washing or dressing yourself and no problems walking about. |

# HTMF, no vent

*You are a parent of someone who has a genetic condition that affects the muscles in his body, leading to muscle wasting that gets worse over time. The condition that your son has is life limiting.* *You are the main carer for your son and he lives at home with you.*

| Domain Name | Description |
| --- | --- |
| Physical: lifting and carrying | You **rarely** need to carry your son as he uses a wheelchair all of the time which he can operate himself. He has very limited movement.  You **very often** need to transfer him (e.g., between his wheelchair, bed, toilet, chair) throughout the day. |
| Physical: pain | You **often** have back and shoulder pain from lifting or carrying your son. |
| Physical: help with self-care | You **always** have to help your son with washing, dressing and using the toilet because of his limited movement.  You **do not need to help** your son eat or carry out activities such as brushing his hair for him as he can raise his hand to his mouth. |
| Physical: sleep | You **often** have to wake up at night to help your son and you **often** wake up in the night worrying about him. |
| Physical: tiredness/exhaustion | You **often** feel physically and mentally tired or exhausted and this affects all areas of your daily life. |
| Impact on time and usual activities | You need to manage your son’s medication and take him to frequent medical appointments. **This takes up a lot of your time. Very often** you do not have time for your usual activities (housework, leisure or family activities) and the things you want to do. |
| Impact on work | You have had to **stop working** entirely to care for your son and this has caused you to **often** worry about your finances. |
| Emotional impact | You feel **very** sad or depressed because of your son’s condition. You find it distressing watching his symptoms get worse. You are **very** worried about your son’s current health and his future because you know his condition will continue to get worse over time. You feel grief for the loss of the life your son could have had. |
| Impact on family relationships | It is **very often** difficult to find time to spend with your other children or your partner due to the amount of time required to care for your son. This causes tension and strain between members of your family. |
| Social impact | You **very often** cancel plans or avoid social events because you are not feeling up to it or you do not have time. |
| Own self-care | You have no problems washing or dressing yourself and no problems walking about. |

# No HTMF, no vent

*You are a parent of someone who has a genetic condition that affects the muscles in his body, leading to muscle wasting that gets worse over time. The condition that your son has is life limiting. You are the main carer for your son and he lives at home with you.*

| Domain Name | Description |
| --- | --- |
| Physical: lifting and carrying | You **rarely** need to carry your son as he uses a wheelchair all of the time. You **have to push** the wheelchair as he cannot operate the wheelchair himself. He has very limited movement.  You **very often** need to transfer him (e.g., between his wheelchair, bed, toilet, chair) throughout the day. |
| Physical: pain | You **often** have back and shoulder pain from lifting or carrying your son. |
| Physical: help with self-care | You need to **wash and dress your son completely** and help him with all aspects of toileting because of his limited movement.  You **need to feed your son** and carry out activities such as brushing his hair for him as he cannot raise his hand to his mouth. |
| Physical: sleep | You **often** have to wake up at night to help your son and you **often** wake up in the night worrying about him. |
| Physical: tiredness/exhaustion | You **often** feel physically and mentally tired or exhausted and this affects all areas of your daily life. |
| Impact on time and usual activities | You need to manage your son’s medication and take him to frequent medical appointments. **This takes up a lot of your time**. **Very often** you do not have time for your usual activities (housework, leisure or family activities) and the things you want to do. |
| Impact on work | You have had to **stop working** entirely to care for your son and this has caused you to **often** worry about your finances. |
| Emotional impact | You feel **very** sad or depressed because of your son’s condition. You find it distressing watching his symptoms get worse. You are **very** worried about your son’s current health and his future because you know his condition will continue to get worse over time. You feel grief for the loss of the life your son could have had. |
| Impact on family relationships | It is **very often** difficult to find time to spend with your other children or your partner due to the amount of time required to care for your son. This causes tension and strain between members of your family. |
| Social impact | You **very often** cancel plans or avoid social events because you are not feeling up to it or you do not have time. |
| Own self-care | You have no problems washing or dressing yourself and no problems walking about. |

# HTMF, night vent

*You are a parent of someone who has a genetic condition that affects the muscles in his body, leading to muscle wasting that gets worse over time. The condition that your son has is life limiting. You are the main carer for your son and he lives at home with you.*

| Domain Name | Description |
| --- | --- |
| Physical: lifting and carrying | You **rarely** need to carry your son as he uses a wheelchair all of the time which he can operate himself. He has very limited movement.  You **very often** need to transfer him (e.g., between his wheelchair, bed, toilet, chair) throughout the day. Other times you use a mechanical hoist. |
| Physical: pain | You **often** have back and shoulder pain from lifting or carrying your son. |
| Physical: help with self-care | You **always** have to help your son with washing, dressing and using the toilet because of his limited movement.  You **do not need to help** your son eat or carry out activities such as brushing his hair for him as he can raise his hand to his mouth. |
| Physical: sleep | You **very** **often** have to wake up at night to help your son and you **often** wake up in the night worrying about him. |
| Physical: tiredness/exhaustion | You **often** feel physically and mentally tired or exhausted and this affects all areas of your daily life. |
| Impact on time and usual activities | You need to manage your son’s medication and take him to frequent medical appointments. **This takes up a lot of your time. Very often** you do not have time for your usual activities (housework, leisure or family activities) and the things you want to do. |
| Impact on work | You have had to **stop working** entirely to care for your son and this has caused you to **often** worry about your finances. |
| Emotional impact | You feel **very** sad or depressed because of your son’s condition. You find it distressing watching his symptoms get worse. You are **very** worried about your son’s current health and his future because you know his condition will continue to get worse over time. You feel grief for the loss of the life your son could have had. |
| Impact on family relationships | It is **very often** difficult to find time to spend with your other children or your partner due to the amount of time required to care for your son. This causes tension and strain between members of your family. |
| Social impact | You **very often** cancel plans or avoid social events because you are not feeling up to it or you do not have time. |
| Own self-care | You have no problems washing or dressing yourself and no problems walking about. |

**No HTMF, night vent**

*You are a parent of someone who has a genetic condition that affects the muscles in his body, leading to muscle wasting that gets worse over time. The condition that your son has is life limiting. You are the main carer for your son and he lives at home with you.*

| Domain Name | Description |
| --- | --- |
| Physical: lifting and carrying | You **rarely** need to carry your son as he uses a wheelchair all of the time. You **have to push** the wheelchair as he cannot operate the wheelchair himself. He has very limited movement.  You **very often** need to transfer him (e.g., between his wheelchair, bed, toilet, chair) throughout the day. Other times you use a mechanical hoist. |
| Physical: pain | You **often** have back and shoulder pain from lifting or carrying your son. |
| Physical: help with self-care | You need to **wash and dress your son completely** and help him with all aspects of toileting because of his limited movement.  You **need to feed your son** and carry out activities such as brushing his hair for him as he cannot raise his hand to his mouth. |
| Physical: sleep | You **very often** have to wake up at night to help your son and you **often** wake up in the night worrying about him. |
| Physical: tiredness/exhaustion | You **often** feel physically and mentally tired or exhausted and this affects all areas of your daily life. |
| Impact on time and usual activities | You need to manage your son’s medication and take him to frequent medical appointments. **This takes up a lot of your time**. **Very often** you do not have time for your usual activities (housework, leisure or family activities) and the things you want to do. |
| Impact on work | You have had to **stop working** entirely to care for your son and this has caused you to **often** worry about your finances. |
| Emotional impact | You feel **very** sad or depressed because of your son’s condition. You find it distressing watching his symptoms get worse. You are **very** worried about your son’s current health and his future because you know his condition will continue to get worse over time. You feel grief for the loss of the life your son could have had. |
| Impact on family relationships | It is **very often** difficult to find time to spend with your other children or your partner due to the amount of time required to care for your son. This causes tension and strain between members of your family. |
| Social impact | You **very often** cancel plans or avoid social events because you are not feeling up to it or you do not have time. |
| Own self-care | You have no problems washing or dressing yourself and no problems walking about. |

**No HTMF, full vent**

*You are a parent of someone who has a genetic condition that affects the muscles in his body, leading to muscle wasting that gets worse over time. The condition that your son has is life limiting. You are the main carer for your son and he lives at home with you.*

| Domain Name | Description |
| --- | --- |
| Physical: lifting and carrying | You **rarely** need to carry your son as he uses a wheelchair all of the time. You **have to push** the wheelchair as he cannot operate the wheelchair himself. He has very limited movement.  You **often** need to transfer him (e.g., between his wheelchair, bed, toilet, chair) throughout the day. Other times you use a mechanical hoist. |
| Physical: pain | You **often** have back and shoulder pain from lifting or carrying your son. |
| Physical: help with self-care | You need to **wash and dress your son completely** and help him with all aspects of toileting because of his limited movement.  You **need to feed your son** and carry out activities such as brushing his hair for him as he cannot raise his hand to his mouth. |
| Physical: sleep | You **very often** have to wake up at night to help your son and you **often** wake up in the night worrying about him. |
| Physical: tiredness/exhaustion | You **often** feel physically and mentally tired or exhausted and this affects all areas of your daily life. |
| Impact on time and usual activities | You need to manage your son’s medication and take him to frequent medical appointments. **This takes up a lot of your time.** **Very often** you do not have time for your usual activities (housework, leisure or family activities) and the things you want to do. |
| Impact on work | You have had to **stop working** entirely to care for your son and this has caused you to **often** worry about your finances. |
| Emotional impact | You feel **very** sad or depressed because of your son’s condition. You find it distressing watching his symptoms get worse. You are **very** worried about your son’s current health and his future because you know his condition will continue to get worse over time. You feel grief for the loss of the life your son could have had. |
| Impact on family relationships | It is **very often** difficult to find time to spend with your other children or your partner due to the amount of time required to care for your son. This causes tension and strain between members of your family. |
| Social impact | You **very often** cancel plans or avoid social events because you are not feeling up to it or you do not have time. |
| Own self-care | You have no problems washing or dressing yourself and no problems walking about. |
