# Supplementary material for: Estimating health-state utility values for family-caregivers of patients with Duchenne muscular dystrophy using time trade-off valuation
Source: J Patient Rep Outcomes. 2026 Apr 10;10:58. doi: 10.1186/s41687-026-01055-8 (PMC13076743; doi:10.1186/s41687-026-01055-8)
Supplement: Supplementary file 2 — Supplementary Material 2 [file 41687_2026_1055_MOESM2_ESM.pdf]

TTO interview script

Interviewer instructions *– shown in* ***italicized and green text****. These are* ***not to be read*** *to the participant.*

Participant instructions *– shown in plain text. These are to be* ***read aloud*** *to the participant.*

Introduction

Good (morning/afternoon/evening), my name is [insert name] and I am a researcher at Vitaccess. Thank you for taking the time to participate in this interview. The aim of this study is to understand the experiences of unpaid family caregivers of people living with a progressive musculoskeletal condition.

During this interview, you will be asked to choose between different descriptions. You have been sent the descriptions prior to the interview. Please have the descriptions in front of you when we conduct the interview as you will be asked to refer to them at different stages. You will be asked to think about how good or bad these descriptions are in your opinion.

Before we begin, there is some information about the interview I would like to share with you:

1. Your feedback will remain anonymous; this means your identity will not be disclosed and all information and comments you provide will remain confidential.

2. Your participation is voluntary, so you can stop participating at any time without giving us a reason.

3. The interview is estimated to take approximately 60 minutes in total to complete.

4. Please take your time when answering the questions. There are no right or wrong answers. We are interested in your opinions. You can also change your mind regarding previous answers you have provided.

5. Please feel free to ask any questions throughout the interview. However, we might have to wait until the end to answer some of them.

6. You will not be told which pharmaceutical company is sponsoring this study or the name of the disease being studied prior to your interview. This is an important part of this study methodology to reduce participant bias. This approach is the standard methodology for conducting this type of study and if you choose to take part, you agree to the sponsor and disease name being withheld until the interview is complete. After the interview is completed, you will be informed of the disease and sponsor company name.

7. If you have any questions during the interview, please just let me know. Do you have any questions before we start?

Background questions

Go through 'Background questionnaire' with participant.

Introduction to health states

The descriptions you are about to read relate to the experiences of unpaid family caregivers of people living with a progressive musculoskeletal condition. We are going to go through a task which we will use to get your views on these descriptions. Before this,

we are going to go through two practice tasks with you. The purpose of these are to make sure you understand the descriptions so please ask me if you have any questions as we go through them.

Ranking exercise

Go to health state ranking exercise slide in the accompanying slide deck.

I would now like you to read the descriptions, one at a time, and tell me where you would rank them from best to worst. You can change your mind about the placement of each description as you are working through this exercise.

Give participant time to read through the first health state in full.

Where would you place this health state from best to worst?

Adjust health state symbol on the slide accordingly.

Repeat process for remaining health states.

Are you happy with the final ranking of the descriptions?

Once participant confirms they are happy with the final ranking of the health states, the task is complete. Record health state ranking in the accompanying data entry sheet.

Rating exercise

Go to health state rating exercise slide in the accompanying slide deck.

For this next task, we are going to use the scale – as shown on the screen now – to get your opinions on how good or bad you think each of the descriptions is.

The worst or least preferred descriptions would be down towards 0. The closer you tell me to place the description to 0 the worst you think it is.

The best or most preferred descriptions would be up towards 100. The closer you tell me to place the description to 100 the better you think it is.

To start with, please can you refer to the 'full health' description.

Give participant time to read through the 'full health' health state in full.

This description illustrates 'full health'.

'Full health' is equal to 100 on the scale and it is placed at 100. This is the best the health can be.

Please can you now refer to the 'dead' description.

Give participant time to read through the 'dead' health state in full.

Where would you place the dead description on the scale you see?

Adjust health state symbol on the slide accordingly.

If you would like to move the dead description at any point in the future, please let me know.

I will now ask you to read through the remaining descriptions, one at a time. Please read each description carefully and think about how good or bad you think it is.

After you have finished reading it, I would like you to let me know where to place it on the scale depending on where you think it belongs; remembering 100 is the most preferred description and 0 is the least preferred description.

You can also decide that some descriptions are worse than being dead, and you can place it at a lower point down the scale.

When you read the descriptions, imagine living as the person in the description for the rest of your life.

You can ask me to move the descriptions around on the scale if you wish to.

Let's start with the description with the symbol *[say name of symbol]*.

Give participant time to read through the health state in full.

Where would you place this description on the scale you see?

Adjust health state symbol on the slide accordingly.

Repeat process for remaining health states.

Some participants may interpret the scale the wrong way round by placing the worst health states towards 100. If you suspect they are doing this, then check with them (e.g., have them confirm that they believe the states are close to 'full health' or 'dead' as appropriate). End the interview at the end of the rating task if a participant doesn't realise their mistake. Be wary of guiding participants or suggesting to them that their answers are in any way incorrect.

Now that you have completed this task, would you like to make any changes to where you have placed them?

If participant has indicated any health states as worse than dead, confirm this is correct with them.

I would now like to record your values for each description on the scale.

Starting at the bottom of the scale, please read off the value on the scale that you have given to that vignette.

Record health state rating responses in the accompanying data entry sheet.

We are now going to move to the next part of the interview. We will ask you to rate the same descriptions but using a different method.

TTO exercise

Go to TTO exercise slide in the accompanying slide deck.

I will now present you with a series of two choices. I will ask you to choose the one you prefer. If you think the two choices are about the same, let me know and I will note this down. I will be using a visual aid to help with this task.

Move to first TTO exercise board slide in the accompanying slide deck.

The scale beside each vignette represents the period of time you can expect to live in this state for. For the purpose of this exercise, please imagine that the longest that you

can expect to live is 10 years. Each scale will also show the number of years of life lost due to an early death.

Point to the yellow scale.

The yellow colour shows the number of years of full health.

In life A, you would live 10 years, from today, after that you would die.

Point to the navy scale.

The navy colour represents the time you will live in Life B. Think about how good or bad Life B would be for you. In life B, you can expect to live a life as described on the description and you will live for 10 years, from today, followed by death.

Do you understand these ideas?

If no, repeat the explanation.

To start with, please can you refer to the 'full health' description.

Give participant time to read through the 'full health' health state in full.

The duration of time spent in this 'full health' is represented by yellow on the scale, this is Life A.

Please can you now refer to the 'dead' description.

Give participant time to read through the 'dead' health state in full.

Please now consider this description to be Life B.

Would you prefer to live for 10 years in Life A or 10 years in Life B, or are they about the same?

If participant answered Life B or about the same, check that they understand how they have answered.

If repeating the explanation and practice exercise does not improve participant's understanding, or the participant seems uninterested, terminate the interview.

We will now go through this task for the remaining descriptions.

Let's start with the description with the symbol *[say name of symbol]*.

Give participant time to read through the health state in full.

As a reminder, the top part of the board represents Life A. This is time spent in 'full health'. The scale shows that 'full health' will last for 10 years, followed by death. The bottom part of the board represents Life B. This is time spent living in the description of health you have just read. The bottom scale shows that this state will last for 10 years, followed by death.

1. Which life would you prefer, 10 years in life A or 10 years in life B, or are they about the same?

If participant is unsure at any stage:

Would you like me to explain this again?

A – go to life A scale with 0 years (all blank) and continue
B – ask "why?" Mark response (prefer B: 1.0, and note reason given)
Same – mark response (equal: 1.00)

2. Now I've changed the Life A time scale to 0 which can be considered as 'dead'. Which Life would you prefer? 10 years in Life B or being 'dead'?

A or same – go to lead-time TTO interview guide
B – go to life A with 9.5 years and continue

3. Now Life A represents 9 years and 6 months of 'full health' in total, which Life would you prefer? Or are they about the same?

A – go to life A scale with 0.5 years,
B – mark response (prefer B: 0.975) go to the next health state
Same – mark response (equal: 0.950)

4. Now you have 6 months of 'full health' followed by death in Life A, which Life would you prefer? Or are they about the same?

A – mark response (prefer A: 0.025), go to the next health state
B – go to life A scale with 9 years and continue
Same – mark response (equal: 0.050)

5. Now you have 9 years of 'full health' followed by death in Life A, which Life would you prefer? Or are they about the same?

A – go to life A scale with 1 year and continue
B – mark response (prefer B: 0.925) go to the next health state
Same – mark response (equal: 0.900)

6. Now you have 1 year of 'full health' followed by death in Life A, which Life would you prefer? Or are they about the same?

A – mark response (prefer A: 0.075), go to the next health state
B – go to life A scale with 8.5 years and continue
Same – mark response (equal: 0.100)

7. Now you have 8 years and 6 months of 'full health' followed by death in Life A, which Life would you prefer? Or are they about the same?

A – go to life A scale with 1.5 years and continue
B – mark response (prefer B: 0.875) go to the next health state
Same – mark response (equal: 0.850)

8. Now you have 1 year and 6 months of 'full health' followed by death in Life A, which Life would you prefer? Or are they about the same?

A – mark response (prefer A: 0.125), go to the next health state
B – go to life A scale with 8 years and continue
Same – mark response (equal: 0.150)

9. Now you have 8 years of 'full health' followed by death in Life A, which Life would you prefer? Or are they about the same?

A – go to life A scale with 2 years
B – mark response (prefer B: 0.825) go to the next health state
Same – mark response (equal: 0.800)

10. Now you have 2 of 'full health' followed by death in Life A, which Life would you prefer? Or are they about the same?

A – mark response (prefer A: 0.175), go to the next health state
B – go to life A scale with 7.5 years and continue
Same – mark response (equal: 0.200)

11. Now you have 7 years and 6 months of 'full health' followed by death in Life A, which Life would you prefer? Or are they about the same?

A – go to life A scale with 2.5 years
B – mark response (prefer B: 0.775) go to the next health state
If health states same – mark response (equal: 0.750)

12. Now you have 2 years and 6 months of 'full health' followed by death in Life A, which Life would you prefer? Or are they about the same?

A – mark response (prefer A: 0.225), go to the next health state
B – go to life A scale with 7 years and continue
Same – mark response (equal: 0.250)

13. Now you have 7 years of 'full health' followed by death in Life A, which Life would you prefer? Or are they about the same?

A – go to life A scale with 3 years
B – mark response (prefer B: 0.725) go to the next health state
Same – mark response (equal: 0.700)

14. Now you have 3 years of 'full health' followed by death in Life A, which Life would you prefer? Or are they about the same?

A – mark response (prefer A: 0.275), go to the next health state
B – go to life A scale with 6.5 years and continue
Same – mark response (equal: 0.300)

15. Now you have 6 years and 6 months of 'full health' followed by death in Life A, which Life would you prefer? Or are they about the same?

A – go to life A scale with 3.5 years and continue
B – mark response (prefer B: 0.675) go to the next health state
Same – mark response (equal: 0.650)

16. Now you have 3 years and 6 months of 'full health' followed by death in Life A, which Life would you prefer? Or are they about the same?

A – mark response (prefer A: 0.325), go to the next health state
B – go to life A scale with 6 years and continue
Same – mark response (equal: 0.350)

17. Now you have 6 years of 'full health' followed by death in Life A, which Life would you prefer? Or are they about the same?

A – go to life A scale with 4 years
B – mark response (prefer B: 0.625) go to the next health state
Same – mark response (equal: 0.600)

18. Now you have 4 years of 'full health' followed by death in Life A, which Life would you prefer? Or are they about the same?

A – mark response (prefer A: 0.375), go to the next health state
B – go to life A scale with 5.5 years and continue
Same – mark response (equal: 0.400)

19. Now you have 5 years and 6 months of 'full health' followed by death in Life A, which Life would you prefer? Or are they about the same?

A – go to life A scale with 4.5 years
B – mark response (prefer B: 0.575) go to the next health state
Same – mark response (equal: 0.550)

20. Now you have 4 years and 6 months of 'full health' followed by death in Life A, which Life would you prefer? Or are they about the same?

A – mark response (prefer A: 0.425), go to the next health state
B – go to life A scale with 5 years and continue
Same – mark response (equal: 0.450)

21. Now you have 5 years of 'full health' followed by death in Life A, which Life would you prefer? Or are they about the same?

A – mark response (prefer A: 0.475) go to the next health state
B – mark response (prefer B: 0.525), go to the next health state
Same – mark response (equal: 0.500)

Lead-TTO exercise

Considering your response to this description I am now going to ask you a bit more about it using a slightly different method.

Go to L-TTO exercise slide in the accompanying slide deck.

On the board you can see two scales both showing 20 years. The top scale is labelled Life A and the bottom scale is labelled Life B. As before, we want to know which you prefer, imagining that you are the individual described.

Please imagine that in the top scale, Life A, you would live for 10 years, from today, in the yellow 'full health' state, and then you would die. In the bottom scale, Life B, you would live for 10 years, from today, in the yellow 'full health' state, followed by 10 years as the person described in the description you have in front of you, and then you would die.

Do you understand these ideas?

Go to life A scale with 10 years.

1. Now, would you prefer Life A, Life B, or are they about the same?

A – go to life A scale with 0 years and continue
B – mark response (prefer B: 0.0) go to the next health state (return to TTO exercise)

Same – mark response (equal: 0.0)

2. Now I've changed Life A to 0 which can be considered as 'dead', which Life would you prefer or are they about the same?

A – mark response (prefer A: -1.0) go to the next health state (return to TTO exercise)
B – go to life A scale with 9.5 years and continue
Same – mark response (equal: -1.000)

3. Now you have 9 years and 6 months of 'full health' followed by death in Life A, which Life would you prefer or are they about the same?

A – go to life A scale with 0.5 years and continue
B – mark response (prefer B: -0.025) go to the next health state (return to TTO exercise)
Same – mark response (equal: -0.050)

4. Now you have 6 months of 'full health' followed by death in Life A, which Life would you prefer or are they about the same?

A – mark response (prefer A: -0.975) go to the next health state (return to TTO exercise)
B – go to life A scale with 9 years and continue
Same – mark response (equal: -0.950)

5. Now you have 9 years of 'full health' followed by death in Life A, which Life would you prefer or are they about the same?

A – go to life A scale with 1 year and continue
B – mark response (prefer b: -0.075) go to the next health state (return to TTO exercise)
Same – mark response (equal: -0.100)

6. Now you have 1 year of 'full health' followed by death in Life A, which Life would you prefer or are they about the same?

A – mark response (prefer A: -0.925) go to the next health state (return to TTO exercise)
B – go to life A scale with 8.5 years and continue
Same – mark response (equal: -0.900)

7. Now you have 8 years and 6 months of 'full health' followed by death in Life A, which Life would you prefer, or are they about the same?

A – go to life A scale with 1.5 years and continue
B – mark response (prefer B: -0.125) go to the next health state (return to TTO exercise)
Same – mark response (equal: -0.150)

8. Now you have 1 year and 6 months of 'full health' followed by death in Life A, which Life would you prefer, or are they about the same?

A – mark response (prefer A: -0.875) go to the next health state (return to TTO exercise)
B – go to life A scale with 8 years and continue
Same – mark response (equal: -0.850)

9. Now you have 8 years of 'full health' followed by death in Life A, which Life would you prefer or are they about the same?

A – go to life A scale with 2 years and continue
B – mark response (prefer B: -0.175) go to the next health state (return to TTO exercise)
Same – mark response (equal: -0.200)

10. Now you have 2 years of 'full health' followed by death in Life A, which Life would you prefer or are they about the same?

A – mark response (prefer A: -0.825) go to the next health state (return to TTO exercise)
B – go to life A scale with 7.5 years and continue
Same – mark response (equal: -0.800)

11. Now you have 7 years and 6 months of 'full health' followed by death in Life A, which Life would you prefer, or are they about the same?

A – go to life A scale with 2.5 years and continue
B – mark response (prefer B: -0.225) go to the next health state (return to TTO exercise)
Same – mark response (equal: -0.250)

12. Now you have 2 years and 6 months of 'full health' followed by death in Life A, which Life would you prefer, or are they about the same?

A – mark response (prefer A: -0.775) go to the next health state (return to TTO exercise)
B – go to life A scale with 7 years and continue
Same – mark response (equal: -0.750)

13. Now you have 7 years of 'full health' followed by death in Life A, which Life would you prefer, or are they about the same?

A – go to life A scale with 3 years and continue
B – mark response (prefer B: -0.275) go to the next health state (return to TTO exercise)
Same – mark response (equal: -0.300)

14. Now you have 3 years of 'full health' followed by death in Life A, which Life would you prefer, or are they about the same?

A – mark response (prefer A: -0.725) go to the next health state (return to TTO exercise)
B – go to life A scale with 6.5 years and continue
Same – mark response (equal: -0.700)

15. Now you have 6 years and 6 months of 'full health' followed by death in Life A, which Life would you prefer, or are they about the same?

A – go to life A scale with 3.5 years and continue
B – mark response (prefer B: -0.325) go to the next health state (return to TTO exercise)
Same – mark response (equal: -0.350)

16. Now imagine you have 3 years and 6 months of 'full health' followed by death in Life A, which Life would you prefer or are they about the same?

A – mark response (prefer A: -0.675) go to the next health state (return to TTO exercise)

B – go to life A scale with 6 years and continue

Same – mark response (equal: -0.650)

17. Now you have 6 years of 'full health' followed by death in Life A, which Life would you prefer or are they about the same?

A – go to life A scale with 4 years and continue
B – mark response (prefer B: -0.375) go to the next health state (return to TTO exercise)
Same – mark response (equal: -0.400)

18. Now you have 4 years of 'full health' followed by death in Life A, which Life would you prefer or are they about the same?

A – mark response (prefer A: -0.625) go to the next health state (return to TTO exercise)
B – go to life A scale with 5.5 years and continue
Same – mark response (equal: -0.600)

19. Now you have 5 years and 6 months of 'full health' followed by death in Life A, which Life would you prefer, or are they about the same?

A – go to life A scale with 4.5 years and continue
B – mark response (prefer B: -0.425) go to the next health state (return to TTO exercise)
Same – mark response (equal: -0.450)

20. Now you have 4 years and 6 months of 'full health' followed by death in Life A, which Life would you prefer, or are they about the same?

A – mark response (prefer A: -0.575) go to the next health state (return to TTO exercise)
B – go to life A scale with 5 years and continue
Same – mark response (equal: -0.550)

21. Now you have 5 years of 'full health' followed by death in Life A, which Life would you prefer, or are they about the same?

A – mark response (prefer A: -0.525) go to the next health state (return to TTO exercise)
B – mark response (prefer B: -0.475), go to the next health state (return to TTO exercise)
Same – mark response (equal: -0.500) go to the next health state (return to TTO exercise)

At the end of the interview, ask the following questions about any health states valued as worse than dead (L-TTO exercise)

- Are you aware that answer you gave for this description suggests that being the person in this description is worse than being dead?
- Knowing this now, would you change how you evaluated this description?

Note responses in the Notes column on the score sheet for the relevant health state.

Close-out (once all health states have been valuated)

We have now come to the end of the interview.

As discussed earlier, I can now let you know that the sponsor of this study is the pharmaceutical company Pfizer and the disease descriptions you were asked to read were of caregivers of people with Duchenne muscular dystrophy, a rare genetic disorder.

Thank you very much for your participation. You will receive your voucher as a thank you for your time participating in these activities via email within two weeks.
