# Supplementary material for: Estimating health-state utility values for family-caregivers of patients with Duchenne muscular dystrophy using time trade-off valuation
Source: J Patient Rep Outcomes. 2026 Apr 10;10:58. doi: 10.1186/s41687-026-01055-8 (PMC13076743; doi:10.1186/s41687-026-01055-8)
Supplement: Supplementary file 4 — Supplementary Material 4 [file 41687_2026_1055_MOESM4_ESM.docx]

Pairwise t-test – TTO scores

Table 1: Adjusted p-values from pairwise within-participant comparisons of TTO scores. Each cell represents the significance of the mean difference between two health states.

| Health State | HS2 | HS3 | HS4 | HS5 | HS6 | HS7 | HS8 |
| --- | --- | --- | --- | --- | --- | --- | --- |
| HS1 | 0.26 | <0.01 | <0.01 | <0.01 | <0.01 | <0.01 | <0.01 |
| HS2 |  | <0.01 | <0.01 | <0.01 | <0.01 | <0.01 | <0.01 |
| HS3 |  |  | 0.99 | 0.37 | <0.01 | <0.01 | <0.01 |
| HS4 |  |  |  | 0.99 | 0.01 | 0.01 | <0.01 |
| HS5 |  |  |  |  | 0.26 | 0.18 | <0.01 |
| HS6 |  |  |  |  |  | 0.99 | 0.6 |
| HS7 |  |  |  |  |  |  | 0.42 |
| P-values were obtained from pairwise paired t-tests conducted on within‑participant differences. All tests were two‑sided with α = 0.05, and Holm adjustment was applied to control the family‑wise error rate across all pairwise comparisons. HS = health state. | | | | | | | |
