# Supplementary figures and images for: Estimating health-state utility values for family-caregivers of patients with Duchenne muscular dystrophy using time trade-off valuation
Source: J Patient Rep Outcomes. 2026 Apr 10;10:58. doi: 10.1186/s41687-026-01055-8 (PMC13076743; doi:10.1186/s41687-026-01055-8)

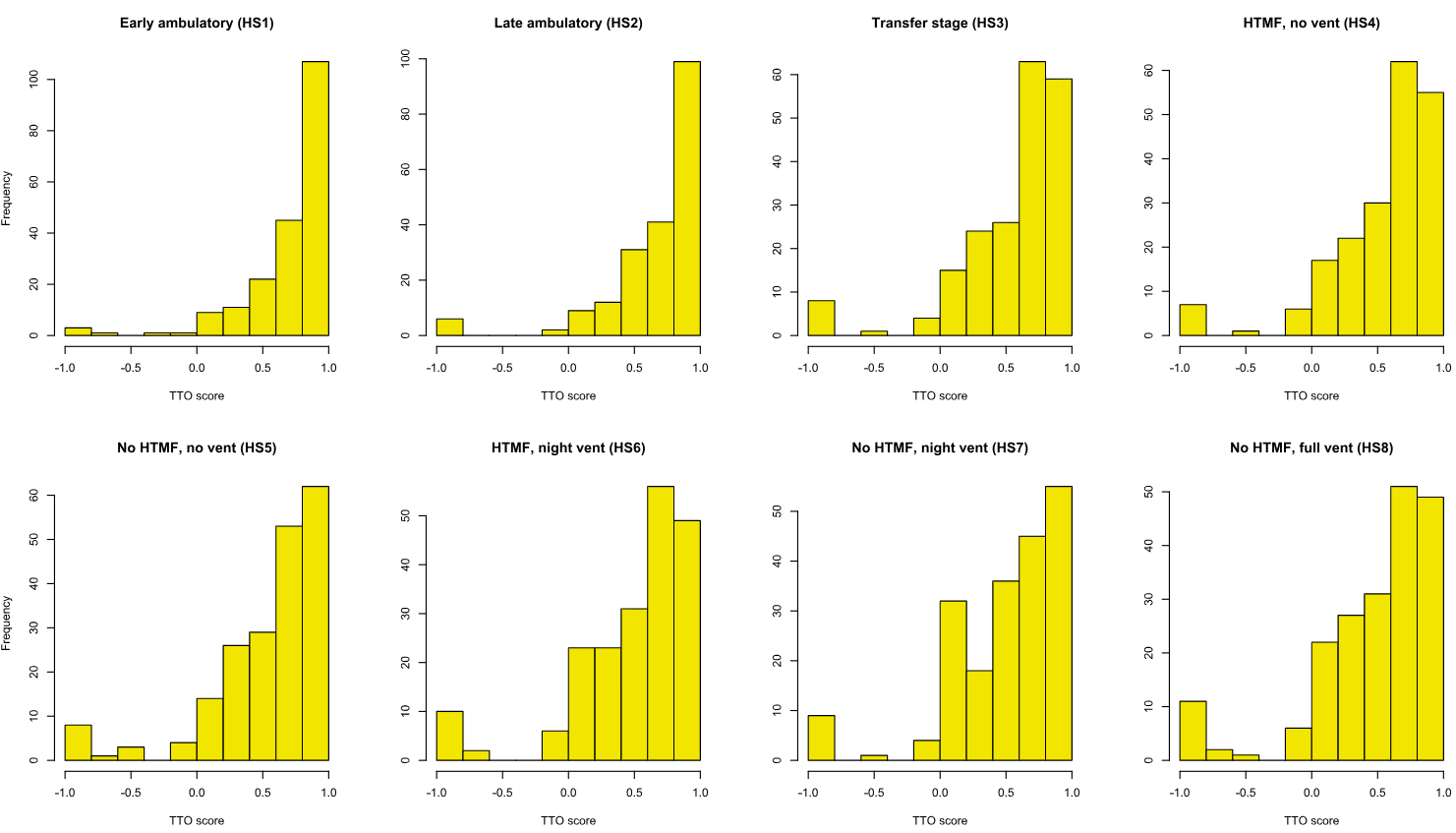

Supplement: Supplementary file 5 — Supplementary Material 5 [file 41687_2026_1055_MOESM5_ESM.png]
